# Differential Tolerance to Direct and Indirect Density-Dependent Costs of Viral Infection in *Arabidopsis thaliana*

**DOI:** 10.1371/journal.ppat.1000531

**Published:** 2009-07-31

**Authors:** Israel Pagán, Carlos Alonso-Blanco, Fernando García-Arenal

**Affiliations:** 1 Centro de Biotecnología y Genómica de Plantas (UPM-INIA) and E.T.S.I. Agrónomos, Campus Montegancedo, Universidad Politécnica de Madrid, Pozuelo de Alarcón (Madrid), Spain; 2 Departamento de Genética Molecular de Plantas, Centro Nacional de Biotecnología, Consejo Superior de Investigaciones Científicas (CNB-CSIC), Darwin 3, Campus Universidad Autónoma, Cantoblanco, Madrid, Spain; University of Basel, Switzerland

## Abstract

Population density and costs of parasite infection may condition the capacity of organisms to grow, survive and reproduce, *i.e.* their competitive ability. In host–parasite systems there are different competitive interactions: among uninfected hosts, among infected hosts, and between uninfected and infected hosts. Consequently, parasite infection results in a direct cost, due to parasitism itself, and in an indirect cost, due to modification of the competitive ability of the infected host. Theory predicts that host fitness reduction will be higher under the combined effects of costs of parasitism and competition than under each factor separately. However, experimental support for this prediction is scarce, and derives mostly from animal–parasite systems. We have analysed the interaction between parasite infection and plant density using the plant-parasite system of *Arabidopsis thaliana* and the generalist virus *Cucumber mosaic virus* (CMV). Plants of three wild genotypes grown at different densities were infected by CMV at various prevalences, and the effects of infection on plant growth and reproduction were quantified. Results demonstrate that the combined effects of host density and parasite infection may result either in a reduction or in an increase of the competitive ability of the host. The two genotypes investing a higher proportion of resources to reproduction showed tolerance to the direct cost of infection, while the genotype investing a higher proportion of resources to growth showed tolerance to the indirect cost of infection. Our findings show that the outcome of the interaction between host density and parasitism depends on the host genotype, which determines the plasticity of life-history traits and consequently, the host capacity to develop different tolerance mechanisms to the direct or indirect costs of parasitism. These results indicate the high relevance of host density and parasitism in determining the competitive ability of a plant, and stress the need to simultaneously consider both factors to understand the selective pressures that drive host–parasite co-evolution.

## Author Summary

Parasites are a potent selective force, as they reduce the fitness of their hosts through a direct cost of infection, due to parasitism itself, and an indirect cost, due to modification of the competitive ability of the infected host. Theory predicts that fitness reduction will be higher under the combined effects of costs of parasitism and host population density than under each factor separately, but experimental support for this prediction is scarce, and derives mostly from animal–parasite systems. We have analysed the combined effects of host density and costs of infection using the plant virus *Cucumber mosaic virus* and its host plant *Arabidopsis thaliana*. The interaction between these factors may result in a reduction or an increase of plant competitive ability, depending on host genotype, which determines the plasticity of life-history traits and consequently, different tolerance mechanisms to the combined effects of plant density and direct or indirect cost of parasitism. These tolerance mechanisms are associated with resource reallocation. Our results stress the relevance of the interaction between host and parasite traits in determining the outcome of infection contributing to understand the selective pressures that drive host–parasite co-evolution.

## Introduction

Competition is one of the major selection factors in nature, acting at all phases of development [1]. The key role of competition in shaping evolution is one of the bases of the Darwinian Theory, underlying the colonization success, expansion and suppression of genotypes and species. The relevance of competition for ecology and evolutionary biology has lead to a large body of work based on life-history theory, which states that competitive ability depends on trade-offs between the capacity of an organism to grow, survive and reproduce [2],[3]. The optimal amount of resources allocated to each of these components may be modified depending on environmental conditions in order to maximize the organism's fitness [4]. Experimental analyses have shown that competition due to increased population density may induce severe alterations in life history traits that are components of competitive ability such as mortality rate [5], time span to maturity [6], adult size [7] or fecundity [8].

The impact of population density on the effects of predation and herbivory have been widely investigated [9]–[11]. In contrast, density dependent effects on host-parasite systems, have received much less attention [12]. In host-parasite systems there are different competitive interactions: intra-class competition among uninfected hosts or among infected hosts, and inter-class competition between uninfected and infected hosts. Each interaction may have different effects on host life-history traits, resulting in a direct cost of infection, due to parasitism itself, and in an indirect cost, due to modification of the competitive ability of the infected host, both being modulated by host population density [13]. Theory predicts that fitness reduction will be higher under the combined effects of host population density and parasitism than under each factor separately [14]–[17]. The outcome of this interaction may also depend on the prevalence of infection, the effect on the host competitive ability being less severe as prevalence increases [13]. Experimental analyses of these predictions derive mostly from animal-parasite systems [18]–[22], and few similar ones have been carried out with plants [23]–[26].

Furthermore, hosts have evolved defences against parasites, including tolerance mechanisms. Here, we define tolerance as the host ability to reduce the effect of infection on its fitness [27]–[29]. Theoretical and experimental analyses support that tolerance involves modification of life-history traits in order to maximize progeny production through resource reallocation from growth to reproductive structures [4],[27],[30]. In plants, tolerance has been shown to act in response to the combined effects of increasing population density and herbivory [10], but its role under the combined effects of host population density and parasitism has not been previously analysed. Thus, the interaction between parasitism and host density remains largely an unexplored aspect of the evolutionary ecology of parasites that requires further experimentation with a larger array of systems.

We have addressed this question in a plant-virus system, using the widespread virus *Cucumber mosaic virus* (CMV-*Bromoviridae*), a generalist parasite and an important plant pathogen [31], and its host plant *Arabidopsis thaliana* L. (Heynh.) (*Brassicaceae*) (from here on, *Arabidopsis*), a model organism for molecular plant genetics and, more recently, for plant-parasite co-evolution [30],[32],[33]. The capacity of *Arabidopsis* genotypes to modify life-history traits depends on the allometry between vegetative and reproductive organs [30],[33]. Hence, three *Arabidopsis* wild genotypes (referred to as accessions) with different allometry were analysed to estimate costs of CMV infection on life-history traits related to different components of competitive ability at different plant densities and virus prevalence. Our results indicate that the interaction between plant density and costs of infection may result in a reduction or an increase of plant competitive ability. It is shown that host genotype determines the plasticity of life-history traits and consequently, different tolerance mechanisms to the direct or indirect cost of parasitism, which also depend on plant density.

## Results

### Effect of CMV on intra-class competitive ability of *Arabidopsis*: Direct cost of infection

**Figure 1:**
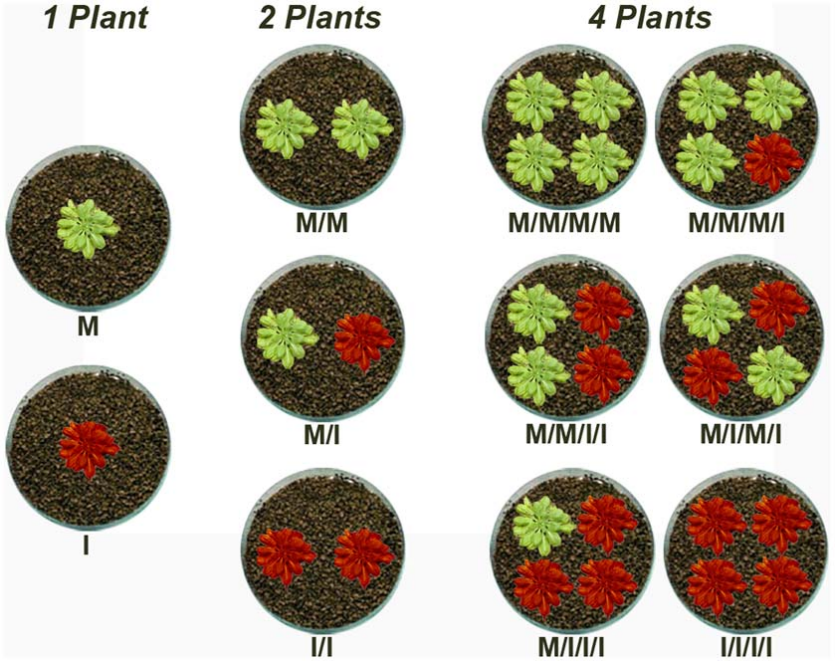
Experimental design used to analyse the combined effects of plant density and costs of infection on competitive ability of *Arabidopsis*. Costs of infection were analysed at 1, 2 and 4 plants per pot using monocultures of infected (I, I/I and I/I/I/I) and mock-inoculated plants (M, M/M and M/M/M/M), as well as in mixed cultures of infected and mock-inoculated plants simulating different CMV prevalence (M/I, M/M/M/I, M/M/I/I and M/I/I/I with infected and mock-inoculated plants next to each other; M/I/M/I with infected and mock-inoculated plants opposite to each other). Fifteen replicated pots per treatment were grown and differences between treatments were analysed by ANOVA.

Since the capacity of *Arabidopsis* genotypes to modify life-history traits depends on the allometry between vegetative and reproductive organs [30],[33], three *Arabidopsis* accessions with different allometry were selected for experimentation. Accessions Cen-1 and L*er*, with a short life cycle and a higher proportion of resources dedicated to reproduction than to growth, and accession Boa-0, with a long life cycle and a higher proportion of resources invested to growth than to reproduction [30]. Individuals of each accession were grown at three different plant densities chosen based on a previous experiment (Fig. S1), which cover from no resource competition to crowded conditions: 1, 2 and 4 plants per pot, arranged in all possible combinations of infected and mock-inoculated plants to simulate different CMV prevalences (Fig. 1). Plants of each accession were inoculated with the LS strain of CMV. The costs of CMV infection on life-history traits related to different components of competitive ability were quantified: rosette weight (*RW*), as a measure of growth effort; inflorescence weight, including seeds, (*IW*) as a measure of total reproductive effort; and seed weight (*SW*), as a measure of progeny production (See Table S1).

**Figure 2:**
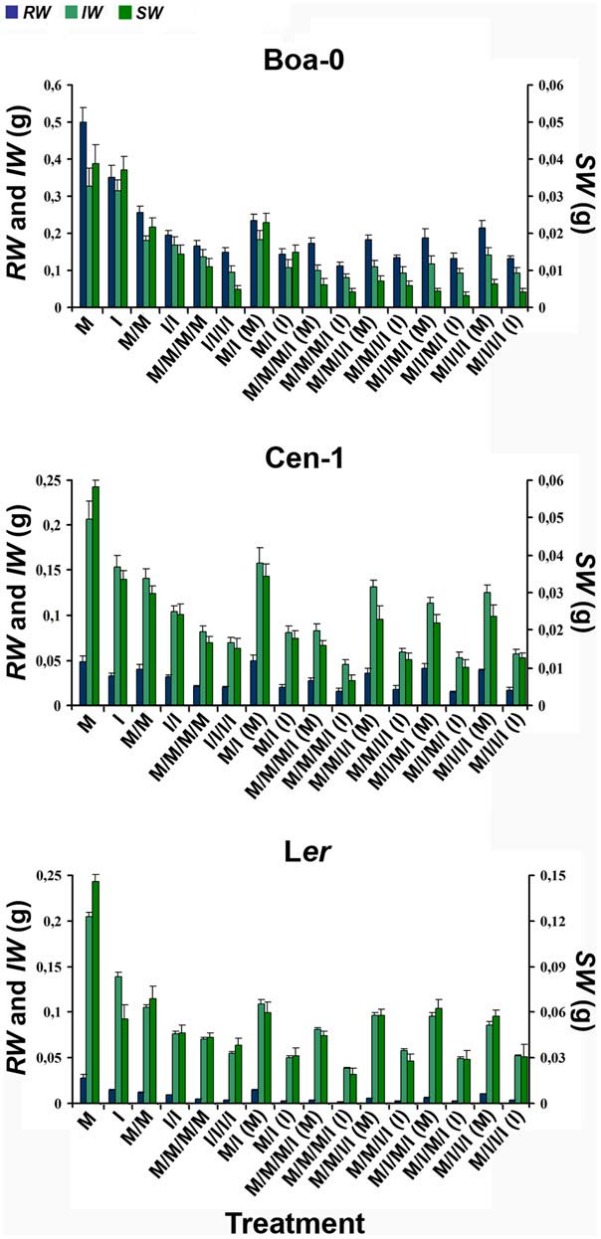
Values of rosette (*RW*), inflorescence (*IW*) and seed (*SW*) weights of *Arabidopsis* accessions Boa-0, Cen-1 and L*er*. Rosettes and inflorescences, including seeds, were weighted as a measurement of vegetative and reproductive efforts, respectively. Total seed weight per plant was used to quantify progeny production. Plants were grown at densities of 1 (M, I), 2 (M/M, I/I, M/I) and 4 (M/M/M/M, I/I/I/I, M/M/M/I, M/M/I/I, M/I/M/I, M/I/I/I) individuals per pot and were infected with CMV according to the experimental design shown in Fig. 1. At 2 and 4 plants per pot, the symbol (M) indicates values for mock-inoculated plants and the symbol (I) values for infected ones. Data are mean±standard errors of trait values derived from 15 pots per treatment. Different scales are used on each panel and on each trait within panels.

The impacts of host population density and viral infection on the competitive ability of *Arabidopsis* were analysed separately. The impact of host population density was measured as the differential performance of *RW*, *IW* and *SW* of plants on monocultures of CMV-infected and on monocultures of mock-inoculated plants at the three plant densities, *i.e.*, in the intra-class treatments I, I/I, I/I/I/I, and M, M/M, M/M/M/M, for 1, 2 and 4 plants per pot, respectively. Two-way ANOVA using plant density and accession as factors showed that in both infected and mock-inoculated plants, the three traits depended on plant density and accession (*F_2,126_*≥13.2, *P*≤1×10^−5^), but only *RW* and *IW* depended on the interaction between both factors (Table S2). Therefore, each accession was analysed separately. In the three accessions, each studied life-history trait was significantly reduced as plant density increased, both for CMV-infected and mock-inoculated plants (*F_2,44_*≥7.6, *P*≤0.002) (Fig. 2 and Table S3). Hence, competition for resources occurred when more than one plant, either mock-inoculated or infected, grew in the same pot, the higher the density, the stronger the decrease of plant growth and progeny production.

The direct cost of CMV infection was determined as the impact of parasitism on intra-class competitive ability comparing plant performance of *RW*, *IW* and *SW* between monocultures of CMV-infected and mock-inoculated plants. All traits differed among plant conditions (infected and mock-inoculated), plant densities and accessions (*F_1,272_*≥11.73, *P*≤7×10^−4^; *F_2,272_*≥63.06, *P*≤1×10^−5^; *F_2,272_*≥41.10, *P*≤1×10^−5^, respectively), interactions being significant for *RW* and *SW*, but not for *IW* (Table S4). Thus, we analysed the direct cost of infection in each plant density and accession separately. For the three accessions, a general reduction of *RW*, *IW* and *SW* was observed at each density for infected plants compared with mock-inoculated ones (*F_1,29_*≥4.37, *P*≤0.046), with the exception of *IW* at 1 and 2 plants per pot, and *SW* at 1 plant per pot in accession Boa-0 (*F_1,29_*≤0.30, *P*≥0.619) (Fig. 2 and Table S5). Thus, accessions Cen-1 and L*er* suffered a direct cost of infection on all traits, both in the absence or presence of competition for resources. In contrast, CMV infection affected the growth (*RW*) of Boa-0 at all densities, but reproductive traits (*IW* and *SW*) of this accession were affected only under severe resource competition.

**Figure 3:**
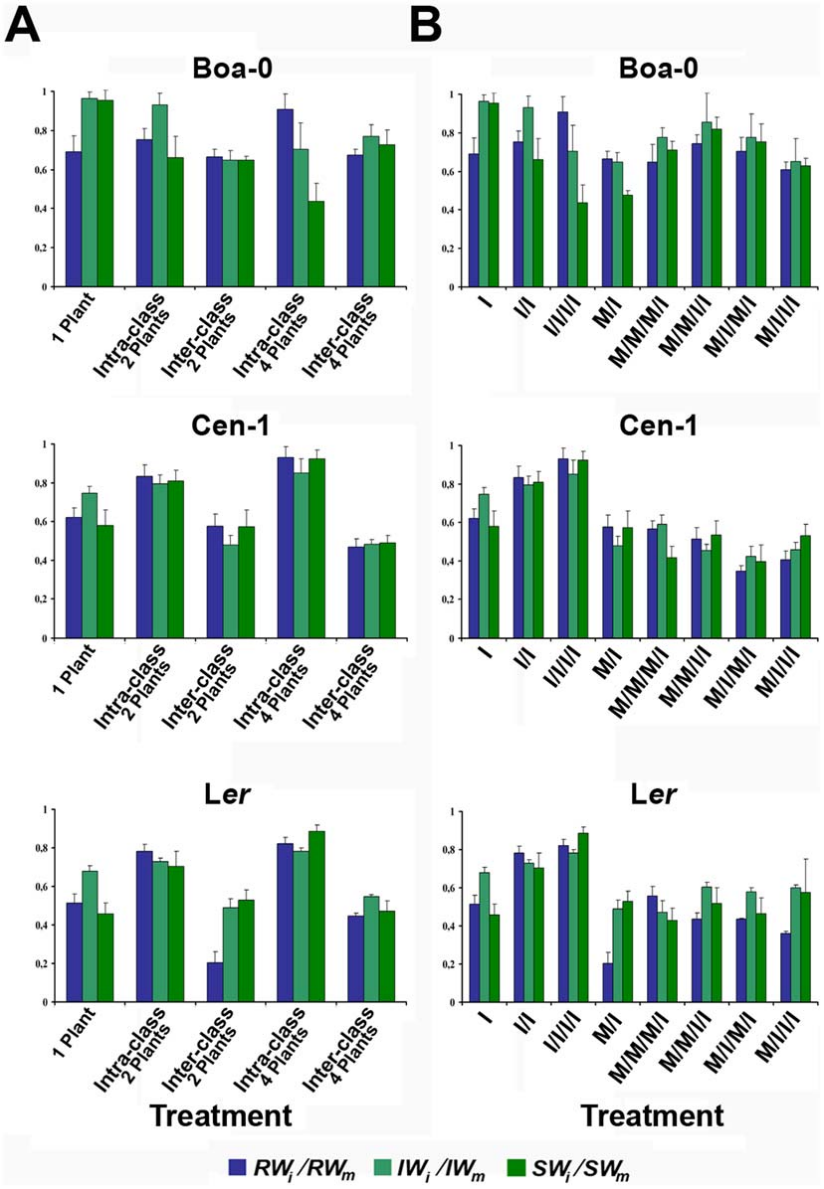
Effect of infection on rosettes (*RW*), inflorescences (*IW*) and seeds (*SW*) weights in intra- and interclass treatments of *Arabidopsis* accessions Boa-0, Cen-1 and L*er*. The effect of virus infection was quantified as the ratio between the values of each trait in infected (*i*) and its mean value in mock-inoculated (*m*) plants (*Trait_i_*/*Trait_m_*). (A) Intra-class values are the ratios between monocultures of infected and mock-inoculated plants (I and M; I/I and M/M; I/I/I/I and M/M/M/M at 1, 2 and 4 plants per pot, respectively). Inter-class values are the mean of the ratios for each inter-class treatment (M/I at 2 plants per pot; M/M/M/I, M/M/I/I, M/I/M/I and M/I/I/I at 4 plants per pot). Data are mean±standard errors from 15 pots for intra-class values and for inter-class values at 2 plants per pot, and from 60 pots for inter-class values at 4 plants per pot. (B) Values of I, I/I and I/I/I/I are the ratios between monocultures of infected and mock-inoculated plants, as in panel A. Values of M/I, M/M/M/I, M/M/I/I, M/I/M/I and M/I/I/I are the corresponding ratios for each inter-class treatment separately. Data are mean±standard errors of trait values from 15 pots per treatment.

The influence of host population density on the direct cost of infection was analysed comparing the effect of infection, defined as the ratio between the value of each trait in infected and in mock-inoculated plants (*Trait_i_*/*Trait_m_*, *i* and *m* denote infected and mock-inoculated plants, respectively), among plant densities (Fig. 3). The effect of infection significantly differed with plant density for all traits (*F_2,126_*≥4.02, *P*≤0.031), but only *SW_i_/SW_m_* differed significantly between accessions (*F_2,126_* = 4.13, *P* = 0.026) (Table S6). In Boa-0 plants, the direct cost of infection on *RW* and *IW* did not significantly differ as plant density increased (*F_2,44_*≤2.33, *P*≥0.108), but it increased for *SW* (*F_2,44_* = 6.89, *P* = 0.002). In Cen-1 and L*er*, direct costs of CMV infection decreased as plant density increased in all traits (*F_2,44_*≥4.88, *P*≤0.027), with the exception of *IW* in Cen-1 (*F_2,44_* = 1.08, *P* = 0.348) (Table S7). Defining tolerance as stated in the introduction, these results indicate that Cen-1 and L*er* increase their tolerance to the direct cost of CMV infection as plant density increases, while the tolerance of Boa-0 decreases when competition occurs.

### Effect of CMV on inter-class competitive ability of *Arabidopsis*: Indirect cost of infection

The indirect cost of infection is determined by the difference between intra- and inter-class competitive ability of infected and non-infected plants [13]. Hence, the performance of infected and mock-inoculated plants was compared between both environments (Fig. 2). *RW*, *IW* and *SW* differed between plant densities and accessions in mock-inoculated and infected plants (*F_1,303_*≥11.42, *P*≤8×10^−4^; *F_2,303_*≥25.65, *P*≤1×10^−5^, respectively), but significant differences between classes of competition were found only for infected plants (*F_1,303_*≥11.08, *P*≤0.001) (Table S8). Hence, analyses were done for each plant density and each accession separately. In the three accessions, the value of *RW*, *IW* and *SW* was similar for mock-inoculated plants from M/I and M/M treatments (*F_1,29_*≤2.51, *P*≥0.124) and was lower for infected plants from M/I than from I/I treatments (*F_1,29_*≥4.64, *P*≤0.047), with the exception of *SW* in Boa-0 (*F_1,29_* = 0.02, *P* = 0.889). At 4 plants per pot, trait values of Boa-0 did not differ between intra- and inter-class treatments of infected plants (*F_4,74_*≤1.50, *P*≥0.210); in mock-inoculated Boa-0 plants *RW* and *IW* values did not differ between classes (*F_4,74_*≤1.17, *P*≥0.329), while *SW* values were higher for M/M/M/M than for the inter-class treatments (*F_4,74_* = 2.99, *P* = 0.024). Mock-inoculated plants of Cen-1 and L*er* showed lower values of all traits for M/M/M/M than for M/M/I/I, M/I/M/I and M/I/I/I, but not than M/M/M/I (*F_4,74_*≥2.80, *P*≤0.033) (Fig. 2). For infected individuals of these accessions, *RW* did not differ between intra and inter-class treatments (*F_4,74_*≤1.18, *P*≥0.327); *IW* and *SW* were higher for I/I/I/I than for M/M/M/I (*F_4,74_*≥2.80, *P*≤0.033), but no differences were found with the other inter-class treatments (Table S1 and Table S9). These results show that there is an indirect cost of CMV infection that depends on host density, CMV prevalence and accession.

Indirect costs of CMV infection were further analysed by comparing the effect of infection (*Trait_i_*/*Trait_m_*) between intra- and inter-class treatments (Fig. 3A). The effect of infection on *RW*, *IW* and *SW* significantly differed according to class of competition and accession (*F_1,303_*≥7.97, *P*≤0.005; *F_2,303_*≥3.33, *P*≤0.037, respectively), but only *RW_i_/RW_m_* depended on plant density (*F_1,303_* = 13.30, *P*≤3×10^−4^). In addition, a significant interaction between these three factors was detected on all traits (*F_2,303_*≥2.93, *P*≤0.049) (Table S10). Therefore, the effect of infection was analysed for each accession at each plant density separately. The effect of infection on Boa-0 plants differed depending on the trait: for *RW* it was higher for inter- than for intra-class treatments at both plant densities (*F_1,29_* = 3.63, *P* = 0.043; *F_1,74_* = 10.72, *P* = 0.002, for 2 and 4 plants per pot, respectively); for *IW* it was higher for inter- than for intra-class treatments at 2 plants per pot (*F_1,29_* = 4.88, *P* = 0.037), but not at 4 plants per pot (*F_1,74_* = 0.25, *P* = 0.619, respectively); and for *SW* no difference was found at 2 plants per pot (*F_1,29_* = 0.01, *P* = 0.931), but the effect of infection was higher on intra- than on inter-class treatments at 4 plants per pot (*F_1,74_* = 9.52, *P* = 0.004) (Fig. 3A). The effect of infection on Cen-1 and L*er* plants was higher on inter- than on intra-class treatments for all traits at both plant densities (*F_1,29_*≥6.36, *P*≤0.023; *F_1,74_*≥9.67, *P*≤0.003 for 2 and 4 plants per pot, respectively) (Fig. 3A and Table S11). Thus, in accessions Cen-1 and L*er* there is an indirect cost of CMV infection on both growth and reproductive traits. In contrast, accession Boa-0 did not show costs on reproductive traits at high plant density, which indicates an increased tolerance to the indirect cost of infection.

Host density dependence of the indirect cost of infection was analysed by quantifying the ratio between the effect of infection on inter-class competition and on intra-class competition treatments [(*Trait_i_*/*Trait_m_*)*_Inter-class_*/(*Trait_i_*/*Trait_m_*)*_Intra-class_*] (Fig. 3A). For all traits, this ratio significantly depended on the accession (*F_2,219_*≥4.63, *P*≤0.011), but not on the plant density (*F_1,219_*≤3.38, *P*≥0.067). However, the interaction between both factors was significant (*F_2,219_*≥5.17, *P*≤0.006), indicating that the effect of plant density in the indirect cost of infection differed between accessions (Table S12). Hence, the ratio between the effect of infection on inter-class competition and intra-class competition treatments was analysed for each accession separately. In Boa-0, the indirect cost of infection on *RW* did not differ between plant densities (*F_1,74_* = 0.78, *P* = 0.379). However, for *IW* it was higher at 2 than at 4 plants, while for *SW* it was higher at 2 than at 4 plants per pot (*F_1,74_*≥4.33, *P*≤0.041). In Cen-1 and L*er*, the indirect cost of infection on *IW* did not differ between plant densities (*F_1,74_*≤2.39, *P*≥0.127), while on *RW* it was higher at 4 plants per pot in Cen-1 (*F_1,74_* = 4.18, *P* = 0.040), and at 2 plants per pot in L*er* (*F_1,74_* = 55.54, *P* = 1×10^−5^). The indirect cost on *SW* was higher at 4 plants per pot for both accessions (*F_1,74_*≥4.57, *P*≤0.039) (Fig. 3A and Table S13). Similar results were obtained when treatments with the same prevalence at 2 and 4 plants per pot were compared (M/I, and M/M/I/I or M/I/M/I). Hence, plant density affects the indirect cost of infection, and may increase or decrease such cost depending on host genotype.

The effect of parasite prevalence on the indirect cost of infection was analysed by comparing the effect of infection (*Trait_i_*/*Trait_m_*) between the intra-class treatment and the various inter-class treatments at 4 plants per pot (Fig. 3B). Indirect cost of infection depended on the CMV prevalence and the accession for *RW* and *IW* (*F_4,210_*≥3.14, *P*≤0.016; *F_2,210_*≥8.94, *P*≤2×10^−4^, for prevalence and accession, respectively), but not for *SW* (*F_4,210_*≤1.57, *P*≥0.184; *F_2,219_*≤1.36, *P*≥0.259, respectively), the interaction being significant for all traits (*F_8,219_*≥2.12, *P*≤0.045; Table S14). Hence, we analysed the effect of parasite prevalence for each accession separately. In Boa-0, the indirect cost of infection did not differ between interclass treatments for *RW* and *IW* (*F_4,74_*≤2.17, *P*≥0.113), and it was higher in M/I/I/I than in the rest of inter-class treatments for *SW* (*F_4,74_* = 4.17, *P* = 0.004). In Cen-1 and L*er*, indirect costs on *RW* increased as prevalence increased (*F_4,74_*≥7.63, *P*≤1×10^−5^), and costs in *SW* did not differ among inter-class treatments (*F_4,74_*≥2.43, *P*≤0.056). In Cen-1 the indirect cost of infection on *IW* was lowest at the lowest prevalence, while in L*er* it was highest at the lowest prevalence (*F_4,74_*≥9.24, *P*≤1×10^−5^). Hence, in all accessions prevalence differentially affected the indirect cost of infection on each trait, seed production only being affected in Boa-0 plants, where high prevalence of infection reduced tolerance (Table S1 and Table S15).

### Tolerance to direct and indirect costs of infection through resource reallocation

**Figure 4:**
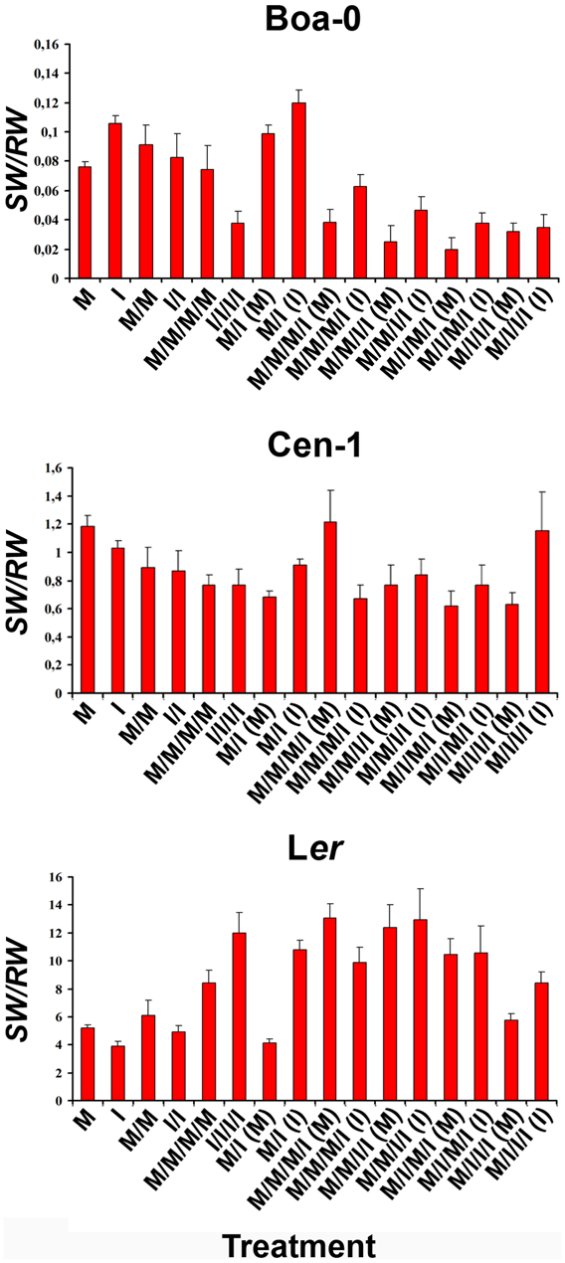
Relationship between resource allocation to progeny production and to growth (*SW/RW*) in *Arabidopsis* accessions Boa-0, Cen-1 and L*er*. Plants were grown at densities of 1 (M, I), 2 (M/M, I/I, M/I) and 4 (M/M/M/M, I/I/I/I, M/M/M/I, M/M/I/I, M/I/M/I, M/I/I/I) individuals per pot and were infected with CMV according to the experimental design shown in Fig. 1. At 2 and 4 plants per pot, the symbol (M) indicates values for mock-inoculated plants and the symbol (I) values for infected ones. Data are mean±standard errors of trait values derived from 15 pots per treatment. Different scales are used on each panel.

Tolerance to CMV infection in *Arabidopsis* under non-competitive conditions is associated with changes in resource allocation patterns [28]. To analyse whether this mechanism is also involved in tolerance to virus infection at increased population density, the relationship between *SW* and *RW* was compared between infected and mock-inoculated plants for each treatment (Fig. 4). The *SW/RW* ratio varied according to plant condition (infected or mock-inoculated), plant density and accession (*F_1,702_*≥3.92, *P*≤0.045; *F_2,702_*≥8.71, *P*≤2×10^−4^; *F_2,702_*≥196.46, *P*≤1×10^−5^) (Table S16). Thus, the *SW/RW* ratio was compared between infected and mock-inoculated plants for each accession at each plant density (Table S17). In intra-class treatments, the *SW/RW* value of Boa-0 was higher on infected than on mock-inoculated plants at 1 plant per pot (*F_1,29_* = 12.87, *P*≤1×10^−5^), but increasingly lower at 2 and 4 plants per pot (*F_1,29_* = 0.16, *P* = 0.696; *F_2,29_* = 18.01, *P*≤1×10^−5,^, for 2 and 4 plants per pot, respectively). In Cen-1 and L*er*, *SW/RW* was higher on mock-inoculated than on infected plants at 1 plant per pot (*F_1,29_*≥6.96, *P*≤1×10^−3^), it was similar at 2 plants per pot (*F_1,29_*≤1.32, *P*≥0.260) and it was higher on infected plants at 4 plants per pot in L*er* (*F_1,29_* = 6.10, *P* = 0.019), but not in Cen-1 (*F_1,29_* = 0.80, *P* = 0.498), (Fig. 4). In inter-class treatments, Boa-0 *SW/RW* value was higher for infected than for mock-inoculated plants for all treatments (*F_1,29_*≥4.62, *P*≤0.035), except for M/I/I/I treatment (*F_1,29_* = 0.06, *P* = 0.802). However, in Cen-1 and L*er* at 2 plants per pot, *SW/RW* was higher on infected than on mock-inoculated plants (*F_1,29_*≥5.97, *P*≤0.021), while at 4 plants per pot this ratio was higher in mock-inoculated than in infected plants for M/M/M/I treatment (*F_1,29_*≥5.88, *P*≤0.024), no differences were observed for M/M/I/I and M/I/M/I (*F_1,29_*≥0.67, *P*≤0.420), and it was higher for infected plants for M/I/I/I treatment (*F_1,29_*≥4.21, *P*≤0.042). Therefore, tolerance to virus infection appears associated with increased resource allocation to seed production, with and without host competition. The degree of this reallocation depended on *Arabidopsis* accession.

## Discussion

Our results demonstrate that the interaction of host population density and parasite infection is highly relevant in determining the competitive ability of a plant. The outcome of this interaction varies for different components of competitive ability, and depends on host genotype and infection prevalence. Thus, in *Arabidopsis* accessions Cen-1 and L*er* that dedicate a higher proportion of resources to reproduction than to growth (Fig. 2), the direct cost of CMV infection decreased as plant density increased, indicating a density-dependent tolerance to CMV infection (Fig. 3). In contrast, these accessions showed a lower level of tolerance to CMV in the absence of competition (Fig. 2, and [30]). The opposite behaviour was observed in accession Boa-0 that invests a higher proportion of resources to growth than to reproduction, since the high tolerance to CMV in the absence of competition (Fig. 2, and [30]) decreased when resource limitation occurred. Boa-0 plants have significantly higher biomass and need more resources to complete their life cycle than Cen-1 or L*er* plants (Fig. 2). Resource availability may limit plant plasticity [34], and the lower amount of resources available under competition may explain the reduced fraction of resources invested in reproduction in infected Boa-0 plants and their reduced tolerance when host density increases. In Cen-1 and L*er*, competition for resources is more intense as population density increases in monocultures of mock-inoculated than of infected plants (Fig. 2). Thus, at higher population densities a larger proportion of resources is available for each infected plant compared with mock-inoculated ones, which may explain their higher plasticity in resource allocation (Fig. 4) and consequently, their tolerance to the direct cost of infection under competition than under unlimited resources. Thus, modification of the resource allocation pattern may partly determine genotype-specific tolerance to the combined effects of plant density and the direct cost of CMV infection, which results in increased competitive ability of Cen-1 and L*er* infected plants, but not of Boa-0. However, factors other than resource allocation between rosette growth and seed production may contribute also to tolerance, as suggested by the observed *SW/RW* differences between Cen-1 and L*er*.

Most experimental studies of host-parasite interactions quantify only the direct cost of infection [35]. Our results indicate that CMV infection has also an indirect cost on *Arabidopsis*, which has evolved genotype-specific tolerance to it. This tolerance suggests that indirect costs are relevant in determining the total costs of parasitism on host fitness, and should be considered for obtaining a realistic evaluation of parasitism. Theory predicts that the indirect cost of infection will depend on the intensity of competition, which is determined by host density and parasite prevalence. It is estimated that the higher the competition intensity, the higher the indirect costs [14]–[16]. In agreement with this prediction, the indirect cost of infection on Cen-1 and L*er* plants increased with plant density, as in most reports on the interactions of plants with parasites or herbivores (e.g. [10],[24],[25],[36]). In contrast, in Boa-0 plants, indirect costs on progeny production disappeared or were overcompensated (infected plants showed a higher inter- than intra-class competitive ability) as plant density increased. As for Cen-1 and L*er* tolerance to the direct cost of infection, tolerance of Boa-0 plants to the indirect cost of CMV infection appears associated with resource reallocation from growth to reproduction (Fig. 4).

The indirect cost of CMV infection was also affected by infection prevalence, but varied depending on the competitive ability component and the plant genotype. In Boa-0, infected plants show a lower intra- than inter-class competitive ability, resulting in a decrease of tolerance as prevalence increases, at odds with theoretical predictions [13]. In contrast, in Cen-1 and L*er* plants, the higher the CMV prevalence, the higher the cost of infection on growth, but no effect of prevalence was observed on seed production. Most reported experimental analyses of competitive ability have focussed on the ability to harvest resources, *i.e.*, growth [9],[37]. In this work, survival and reproduction were also measured, showing their different contribution to competitive success [9],[37]. The differential costs of infection on each life-history trait as prevalence increases indicates that analyses of the costs of parasitism that only consider one trait (e.g., growth) may result in biased conclusions, what underlines the relevance of considering different components of competitive ability to obtain a realistic view of the selection pressures exerted by parasites on their hosts.

In conclusion, plant density and costs of infection shape the competitive ability of plants. The outcome of the interaction between these factors depends on the plant genotype, which determines the plasticity of life-history traits and, hence, tolerance to the combined effects of both factors. Resource reallocation-based tolerance plays a key role in the competitive ability of *Arabidopsis*, which has evolved different strategies to maximize competitiveness in each genotype. Therefore, future analyses should consider not one but all these factors to understand the selective pressures that drive host-parasite co-evolution.

## Materials and Methods

### Viral isolates and *Arabidopsis* accessions

Strain LS-CMV, belonging to subgroup II of CMV isolates, was derived from biologically active cDNA clones [38] by in vitro transcription with T7 RNA polymerase (New England Biolabs, Ipswich MA, USA). Transcripts were used to infect tobacco plants for virus multiplication. CMV virions were purified from infected tobacco leaves as described in [39] and viral RNA was extracted by virion disruption with phenol and sodium dodecyl sulphate.

Three accessions of *Arabidopsis thaliana* were used: Boa-0 (Boadilla, Spain), Cen-1 (Centenera, Spain) and L*er* (Landsberg, Poland). Boa-0 invests a higher proportion of resources to growth than to reproduction, and presents a longer life cycle than Cen-1 and L*er*, which dedicate a higher proportion of resources to reproduction than to growth [30],[33]. The three accessions were multiplied simultaneously in the same greenhouse to obtain the seeds used for the experiments described in this work. Hence, maternal effects were not considered.

### Experimental design

Costs of infection were analysed at 1, 2 and 4 plants per pot using monocultures of infected (I, I/I and I/I/I/I) and mock-inoculated (M, M/M and M/M/M/M) plants, as well as all possible combinations of mixed cultures of infected and mock-inoculated plants, simulating different CMV prevalences (Fig. 1). The following mixed cultures were used: M/I; M/M/M/I; M/M/I/I and M/I/I/I (infected and mock-inoculated plants next to each other); M/I/M/I (infected and mock-inoculated plants opposite to each other). Fifteen replicated pots per treatment were analysed. For plant growth, seeds of each accession were sown on filter paper soaked with water in a single plastic Petri dishes, and stratified in darkness at 4°C for 3 days before transferring for germination to a growth chamber (22°C, 14 h light and 70% relative humidity). Five day-old seedlings were planted in soil containing pots (10.5 cm of diameter and 0.43 l volume) for all plant densities. Plants were grown in a greenhouse (20–25°C day/night, 16 h light) in a completely randomised design. Three rosette leaves per plant were mechanically inoculated with purified CMV RNA (100 ng/µl) in 0.1 M Na_2_HPO_4_ when rosettes presented 4–5 leaves (stages 1.04–1.05 in [40]).

### Quantification of *Arabidopsis* competitive ability traits

Plants were harvested at complete senescence stage, and dry weight was determined after plants were maintained at 65°C until constant weight. The weights of rosettes (rosette weight, *RW*), inflorescence structures including seeds (inflorescence weight, *IW*) and seeds (seed weight, *SW*) were measured separately. Rosette weight was used as an estimate of growth effort, inflorescence weight was taken as an estimate of total reproductive effort (reproductive structures plus seed output). Seed weight was quantified after threshing as a proxy to the number of viable seeds, since CMV infection does not affect either the weight per seed or seed viability in these accessions [31]. Thus, seed weight was used as an estimator of progeny production. To quantify the effect of CMV infection on life history traits under competition (here referred to as competitive ability), the mean value of the infected plants in each pot was divided by the mean value of the mock-inoculated plants of the same treatment (*Trait_i_*/*Trait_m_*, *i* and *m* denote infected and mock-inoculated plants, respectively).

### Statistical analyses

*RW*, *IW* and *SW* and their various transformations, were homocedastic and were analysed using analysis of variance (ANOVA). All the analyses were done using pot as the unit of replication, that is, considering the mean value of each trait for plants of each condition (infected or mock-inoculated) within each pot. All traits were compared among conditions (infected or mock-inoculated), treatments, classes of competition (intra or interclass) or densities by one-way ANOVA. To determine interactions between these factors, complete two-way or three-way ANOVA models were used. Significance of differences among classes within each factor was determined by Least Significant Difference (LSD) analyses. All comparisons were done for the raw untransformed data, and for ratios between values of infected and mock-inoculated plants. All statistical analyses were done using the statistical software package SPSS 13.0 (SPSS Inc., Chicago, USA).

## Supporting Information

Figure S1 Values of rosette (*RW*), inflorescence (*IW*) and seed (*SW*) weights of non-infected Cen-1 plants at four plant densities. To determine the number of plants per pot at which competition for resources occurred, plants were grown at 1, 2, 4 and 6 plants per pot, with five replicates per density. The values of *RW*, *IW* and *SW* decreased as plant density increased in 1, 2 and 4 plants per pot (*F_2,34_*≥3.83, *P*≤0.01), but no differences were found between 4 and 6 plants per pot (*F_1,49_*≤0.24, *P*≥0.63), indicating that competition occurred when more than one plant grew per pot and that crowding conditions were reached at 4 plants per pot.
(0.10 MB TIF)

Table S1 Statistical parameters of virus effects on plant life-history traits.
(0.06 MB PDF)

Table S2 Two-way ANOVAs of *Arabidopsis* life-history traits in infected (I) and mock-inoculated (M) plants, by using “plant density” and “accession” as factors.
(0.03 MB PDF)

Table S3 One-way ANOVAs of the impact of host plant density on *Arabidopsis* life-history traits in infected (I) and mock-inoculated (M) plants.
(0.03 MB PDF)

Table S4 Three-way ANOVAs of life-history traits on *Arabidopsis* monocultures of infected (I) and mock-inoculated (M) plants, by using “plant condition (infected, I or mock-inoculated, M)”, “plant density” and “accession” as factors.
(0.03 MB PDF)

Table S5 One-way ANOVAs of the direct cost of CMV infection on *Arabidopsis* life-history traits. Comparison between monocultures of infected and mock-inoculated plants at each plant density.
(0.03 MB PDF)

Table S6 Two-way ANOVAs of the direct cost of CMV infection (*Trait_i_/Trait_m_*) on *Arabidopsis* life-history traits, by using “plant density” and “accession” as factors.
(0.02 MB PDF)

Table S7 One-way ANOVAs of the impact of host plant density and the direct cost of CMV infection (*Trait_i_/Trait_m_*) on *Arabidopsis* life-history traits.
(0.02 MB PDF)

Table S8 Three-way ANOVAs for the indirect cost of CMV infection in Arabidopsis life-history traits in infected and mock-inoculated plants, by using “class of competition”, “plant density” and “accession” as factors.
(0.04 MB PDF)

Table S9 One-way ANOVAs for the indirect cost of CMV infection on *Arabidopsis* life-history traits in infected and mock-inoculated plants.Comparison between intra and interclass treatments at each plant density.
(0.04 MB PDF)

Table S10 Three-way ANOVAs of the effect of CMV infection (*Trait_i_/Trait_m_*) on *Arabidopsis* life-history traits, by using “class of competition”, “plant density” and “accession” as factors.
(0.03 MB PDF)

Table S11 One-way ANOVAs of the effect of CMV infection (*Trait_i_/Trait_m_*) on *Arabidopsis* life-history traits. Comparison between intra and interclass treatments at each plant density.
(0.03 MB PDF)

Table S12 Two-way ANOVAs of the impact of host plant density in the indirect cost of CMV infection [(*Trait_i_/Trait_m_*)*_Inter-class_*/(*Trait_i_/Trait_m_*)*_Intra-class_*] on *Arabidopsis* life-history, by using “plant density” and “accession” as factors.
(0.02 MB PDF)

Table S13 One-way ANOVAs of the impact of host plant density in the indirect cost of CMV infection [(*Trait_i_/Trait_m_*)*_Inter-class_*/(*Trait_i_/Trait_m_*)*_Intra-class_*] on *Arabidopsis* life-history traits.
(0.03 MB PDF)

Table S14 Two-way ANOVAs of the impact of CMV prevalence at 4 plants per pot in the effect of CMV infection (*Trait_i_/Trait_m_*) on *Arabidopsis* life-history traits, by using “prevalence” and “accession” as factors.
(0.02 MB PDF)

Table S15 One-way ANOVAs of the impact of CMV prevalence at 4 plants per pot in the effect of CMV infection (*Trait_i_/Trait_m_*) on *Arabidopsis* life-history traits.
(0.03 MB PDF)

Table S16 Three-way ANOVAs of *SW/RW* ratio, by using “plant condition (infected, I or mock-inoculated, M)”, “plant density” and “accession” as factors.
(0.02 MB PDF)

Table S17 One-way ANOVAs of *SW/RW* ratio in *Arabidopsis* accessions. Comparison between infected (I) and mock-inoculated (M) plants at each plant density.
(0.03 MB PDF)
