# Supplementary figures and images for: Differential Tolerance to Direct and Indirect Density-Dependent Costs of Viral Infection in Arabidopsis thaliana
Source: PLoS Pathog. 2009 Jul 31;5(7):e1000531. doi: 10.1371/journal.ppat.1000531 (PMC2712083; doi:10.1371/journal.ppat.1000531)

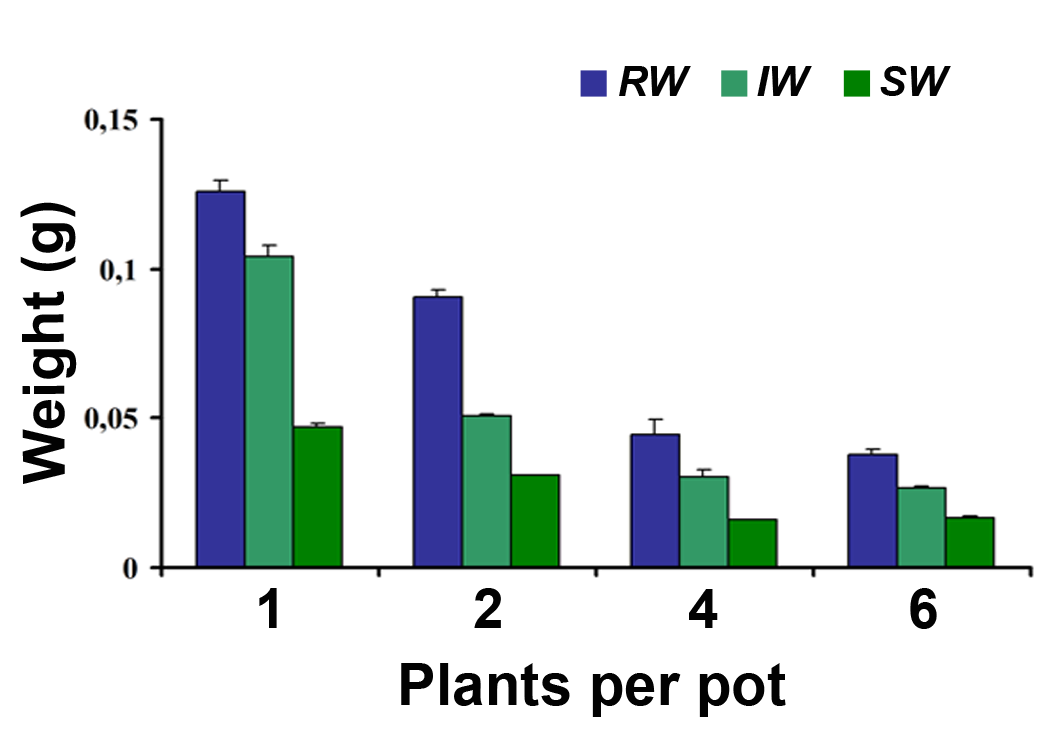

Supplement: Figure S1 — Values of rosette (RW), inflorescence (IW) and seed (SW) weights of non-infected Cen-1 plants at four plant densities. To determine the number of plants per pot at which competition for resources occurred, plants were grown at 1, 2, 4 and 6 plants per pot, with five replicates per density. The values of RW, IW and SW decreased as plant density increased in 1, 2 and 4 plants per pot (F2,34≥3.83, P≤0.01), but no differences were found between 4 and 6 plants per pot (F1,49≤0.24, P≥0.63), indicating that competition occurred when more than one plant grew per pot and that crowding conditions were reached at 4 plants per pot. (0.10 MB TIF) [file ppat.1000531.s001.tif]
