# Supplementary material for: Differential Tolerance to Direct and Indirect Density-Dependent Costs of Viral Infection in Arabidopsis thaliana
Source: PLoS Pathog. 2009 Jul 31;5(7):e1000531. doi: 10.1371/journal.ppat.1000531 (PMC2712083; doi:10.1371/journal.ppat.1000531)
Supplement: Table S1 — Statistical parameters of virus effects on plant life-history traits. (0.06 MB PDF) [file ppat.1000531.s002.pdf]

Table S1. Statistical parameters of virus effects on plant life-history traits.

Accession	Treatment	*RW*	RW_i/RW_m	*IW*	IW_i/IW_m	*SW*	SW_i/SW_m	*SW/RW*
**Boa-0**								
	M	0.500 ± 0.039	-	0.327 ± 0.048	-	0.039 ± 0.005	-	0.083 ± 0.003
	I	0.350 ± 0.032	0.690 ± 0.083	0.314 ± 0.030	0.965 ±0.031	0.037 ± 0.004	0.954 ± 0.051	0.106 ± 0.006
	M/M	0.256 ± 0.017	-	0.180 ± 0.014	-	0.022 ± 0.002	-	0.091 ± 0.014
	I/I	0.194 ± 0.014	0.755 ± 0.053	0.167 ± 0.021	0.931 ±0.059	0.014 ± 0.002	0.662 ± 0.109	0.083 ± 0.016
	M/M/M/M(M)	0.167 ± 0.014	-	0.136 ± 0.020	-	0.011 ± 0.002	-	0.074 ± 0.016
	I/I/I/I(I)	0.149 ± 0.012	0.908 ± 0.080	0.095 ± 0.017	0.705 ±0.134	0.005 ± 0.001	0.425 ± 0,093	0.038 ± 0.009
	M/I(M)	0.234 ± 0.016	-	0.182 ± 0.025	-	0.023 ± 0.003	-	0.099 ± 0.006
	M/I(I)	0.144 ± 0.015	0.666 ± 0.040	0.108 ± 0.023	0.647 ±0.049	0.015 ± 0.002	0.475 ± 0.021	0.120 ± 0.009
	M/M/M/I(M)	0.172 ± 0.016	-	0.101 ± 0.015	-	0.006 ± 0.002	-	0.038 ± 0.009
	M/M/M/I(I)	0.112 ± 0.011	0.649 ± 0.091	0.081 ± 0.010	0.777 ±0.051	0.004 ± 0.001	0.712 ± 0.046	0.063 ± 0.008
	M/M/I/I(M)	0.182 ± 0.013	-	0.109 ± 0.018	-	0.007 ± 0.001	-	0.025 ± 0.011
	M/M/I/I(I)	0.134 ± 0.008	0.742 ± 0.046	0.094 ± 0.016	0.856 ±0.152	0.006 ± 0.001	0.812 ± 0.065	0.047 ± 0.009
	M/I/M/I(M)	0.188 ± 0.023	-	0.118 ± 0.020	-	0.004 ± 0.001	-	0.020 ± 0.008
	M/I/M/I(I)	0.133 ± 0.013	0.705 ± 0.072	0.092 ± 0.014	0.776 ±0.122	0.003 ± 0.001	0.752 ± 0.094	0.038 ± 0.007
	M/I/I/I(M)	0.216 ± 0.019	-	0.141 ± 0.020	-	0.006 ± 0.001	-	0.032 ± 0.006
	M/I/I/I(I)	0.131 ± 0.008	0.609 ± 0.040	0.092 ± 0.016	0.650 ±0.119	0.004 ± 0.001	0.637 ± 0.040	0.035 ± 0.009
Cen-1								
	M	0.049 ± 0.006	-	0.206 ± 0.020	-	0.058 ± 0.002	-	1.184 ± 0.078
	I	0.032 ± 0.003	0.621 ± 0.051	0.154 ± 0.013	0.746 ± 0.035	0.034 ± 0.002	0.580 ± 0.080	1.031 ± 0.054
	M/M	0.041 ± 0.005	-	0.141 ± 0.011	-	0.030 ± 0.002	-	0.894 ± 0.142
	I/I	0.032 ± 0.002	0.832 ± 0.061	0.104 ± 0.006	0.796 ± 0.043	0.024 ± 0.003	0.810 ± 0.055	0.868 ± 0.141
	M/M/M/M(M)	0.022 ± 0.001	-	0.082 ± 0.006	-	0.017 ± 0.002	-	0.770 ± 0.042
	I/I/I/I(I)	0.020 ± 0.001	0.930 ± 0.056	0.070 ± 0.006	0.849 ± 0.74	0.015 ± 0.002	0.923 ± 0.045	0.769 ± 0.044
	M/I(M)	0.050 ± 0.006	-	0.158 ± 0.017	-	0.034 ± 0.003	-	0.682 ± 0.042
	M/I(I)	0.020 ± 0.004	0.577 ± 0.062	0.081 ± 0.007	0.480 ± 0.049	0.018 ± 0.002	0.574 ± 0.084	0.910 ± 0.044
	M/M/M/I(M)	0.028 ± 0.003	-	0.083 ± 0.007	-	0.016 ± 0.001	-	1.211 ± 0.229
	M/M/M/I(I)	0.016 ± 0.004	0.566 ± 0.041	0.045 ± 0.006	0.590 ± 0.048	0.007 ± 0.002	0.469 ± 0.060	0.671 ± 0.098
	M/M/I/I(M)	0.036 ± 0.005	-	0.131 ± 0.008	-	0.023 ± 0.004	-	0.767 ± 0.142
	M/M/I/I(I)	0.018 ± 0.003	0.514 ± 0.058	0.060 ± 0.004	0.455 ± 0.030	0.012 ± 0.002	0.577 ± 0.073	0.836 ± 0.113
	M/I/M/I(M)	0.042 ± 0.005	-	0.113 ± 0.006	-	0.022 ± 0.002	-	0.620 ± 0.104
	M/I/M/I(I)	0.014 ± 0.001	0.346 ± 0.028	0.053 ± 0.006	0.424 ± 0.051	0.010 ± 0.002	0.447 ± 0.089	0.765 ± 0.143
	M/I/I/I(M)	0.039 ± 0.002	-	0.125 ± 0.008	-	0.024 ± 0.003	-	0.628 ± 0.088
	M/I/I/I(I)	0.017 ± 0.002	0.406 ± 0.044	0.057 ± 0.005	0.0460 ± 0.038	0.013 ± 0.001	0.555 ± 0.058	1.151 ± 0.279

Data are mean value of each trait in each accession ± standard error.
RW: Rosette Weight; RW_i/RW_m: Rosette Weight ratio; ***IW***: Inflorescence Weight; IW_i/IW_m: Inflorescence Weight ratio; ***SW***: Seed Weight; SW_i/SW_m: Seed Weight ratio; ***SW/RW***: Seed Weight to Rosette Weight ratio.

Table S1. *Continued…*

Accession	Treatment	*RW*	RW_i/RW_m	*IW*	IW_i/IW_m	*SW*	*V*	*SW/RW*
L*er*								
	M	0.028 ± 0.004	-	0.205 ± 0.005	-	0.146 ± 0.005	-	5.207 ± 0.245
	I	0.014 ± 0.001	0.513 ± 0.048	0.139 ± 0.005	0.680 ± 0.026	0.056 ± 0.009	0.458 ± 0.056	3.878 ± 0.342
	M/M	0.012 ± 0.001	-	0.105 ± 0.002	-	0.069 ± 0.011	-	6.093 ± 1.093
	I/I	0.009 ± 0.001	0.783 ± 0.036	0.076 ± 0.002	0.727 ± 0.019	0.046 ± 0.005	0.703 ± 0.080	4.945 ± 0.422
	M/M/M/M(M)	0.004 ± 0.000	-	0.071 ± 0.002	-	0.043 ± 0.003	-	8.412 ± 0.908
	I/I/I/I(I)	0.003 ± 0.000	0.822 ± 0.031	0.055 ± 0.001	0.781 ± 0.018	0.038 ± 0.006	0.885 ± 0.031	11.984 ± 1.463
	M/I(M)	0.014 ± 0.001	-	0.109 ± 0.005	-	0.059 ± 0.007	-	4.116 ± 0.291
	M/I(I)	0.003 ± 0.000	0.202 ± 0.057	0.050 ± 0.002	0.490 ± 0.045	0.031 ± 0.005	0.528 ± 0.054	10.770 ± 0.690
	M/M/M/I(M)	0.003 ± 0.000	-	0.081 ± 0.002	-	0.044 ± 0.003	-	13.074 ± 0.985
	M/M/M/I(I)	0.002 ± 0.000	0.558 ± 0.048	0.038 ± 0.002	0.473 ± 0.058	0.019 ± 0.004	0.426 ± 0.065	9.919 ± 1.069
	M/M/I/I(M)	0.005 ± 0.000	-	0.096 ± 0.003	-	0.058 ± 0.004	-	12.380 ± 1.664
	M/M/I/I(I)	0.002 ± 0.000	0.435 ± 0.032	0.058 ± 0.002	0.604 ± 0.024	0.028 ± 0.005	0.528 ± 0.083	12.933 ± 2.223
	M/I/M/I(M)	0.006 ± 0.000	-	0.095 ± 0.004	-	0.062 ± 0.005	-	10.461 ± 1.103
	M/I/M/I(I)	0.003 ± 0.000	0.434 ± 0.006	0.050 ± 0.002	0.580 ± 0.019	0.029 ± 0.005	0.434 ± 0.082	10.588 ± 1.883
	M/I/I/I(M)	0.010 ± 0.001	-	0.086 ± 0.003	-	0.057 ± 0.004	-	5.756 ± 0.465
	M/I/I/I(I)	0.004 ± 0.000	0.361 ± 0.011	0.052 ± 0.001	0.600 ± 0.014	0.030 ± 0.010	0.572 ± 0.175	8.447 ± 0.784

Data are mean value of each trait in each accession ± standard error.
RW: Rosette Weight; RW_i/RW_m: Rosette Weight ratio; ***IW***: Inflorescence Weight; IW_i/IW_m: Inflorescence Weight ratio; ***SW***: Seed Weight; SW_i/SW_m: Seed Weight ratio; ***SW/RW***: Seed Weight to Rosette Weight ratio.
