# Supplementary material for: Differential Tolerance to Direct and Indirect Density-Dependent Costs of Viral Infection in Arabidopsis thaliana
Source: PLoS Pathog. 2009 Jul 31;5(7):e1000531. doi: 10.1371/journal.ppat.1000531 (PMC2712083; doi:10.1371/journal.ppat.1000531)
Supplement: Table S2 — Two-way ANOVAs of Arabidopsis life-history traits in infected (I) and mock-inoculated (M) plants, by using “plant density” and “accession” as factors. (0.03 MB PDF) [file ppat.1000531.s003.pdf]

Table S2. Two-way ANOVAs of *Arabidopsis* life-history traits in infected (I) and mock-inoculated (M) plants, by using "plant density" and "accession" as factors.

			**Plant Density**			**Accession**			**D x A**		
Plant Condition	Trait	*n*	*df*	*F*	*P*	*df*	*F*	*P*	*df*	*F*	*P*
Infected											
	RW	135	2	21.5	$1x10^{-5}$	2	232.1	$1x10^{-5}$	4	17.4	$1x10^{-5}$
	IW	135	2	46.7	$1x10^{-5}$	2	34.1	$1x10^{-5}$	4	6.1	$1x10^{-5}$
	SW	135	2	21.8	$1x10^{-5}$	2	36.5	$1x10^{-5}$	4	1.3	0.288
Mock-inoculated											
	RW	135	2	41.9	$1x10^{-5}$	2	267.9	$1x10^{-5}$	4	29.1	$1x10^{-5}$
	IW	135	2	37.7	$1x10^{-5}$	2	13.2	$1x10^{-5}$	4	19.8	$1x10^{-5}$
	SW	135	2	120.6	$1x10^{-5}$	2	154.8	$1x10^{-5}$	4	1.02	0.401

Plant Condition (I or M) and traits (***RW:*** Rosette Weight; ***IW:*** Inflorescence Weight; ***SW:*** Seed Weight) are listed on the left. ***n*****:** number of observations. ***df*****:** degrees of freedom. ***F*****:** *F*-value from the type III sum of squares ANOVA for each factor. ***P:*** Estimated probability of obtaining this *F*-value under the null hypothesis.
