# Supplementary material for: Differential Tolerance to Direct and Indirect Density-Dependent Costs of Viral Infection in Arabidopsis thaliana
Source: PLoS Pathog. 2009 Jul 31;5(7):e1000531. doi: 10.1371/journal.ppat.1000531 (PMC2712083; doi:10.1371/journal.ppat.1000531)
Supplement: Table S3 — One-way ANOVAs of the impact of host plant density on Arabidopsis life-history traits in infected (I) and mock-inoculated (M) plants. (0.03 MB PDF) [file ppat.1000531.s004.pdf]

Table S3. One-way ANOVAs of the impact of host plant density on *Arabidopsis* life-history traits in infected (I) and mock-inoculated (M) plants.

			**Plant Density (I; I/I; I/I/I/I)**			**Plant Density (M; M/M; M/M/M/M)**		
Accession	Trait	*n*	*df*	*F*	*P*	*df*	*F*	*P*
Boa-0								
	RW	45	2	23.8	$1x10^{-5}$	2	40.1	$1x10^{-5}$
	IW	45	2	21.6	$1x10^{-5}$	2	9.6	$3x10^{-4}$
	SW	45	2	30.1	$1x10^{-5}$	2	15.1	$1x10^{-5}$
Cen-1								
	RW	45	2	7.6	0.002	2	9.5	$4x10^{-4}$
	IW	45	2	23.2	$1x10^{-5}$	2	19.7	$1x10^{-5}$
	SW	45	2	12.7	$1x10^{-5}$	2	135.4	$1x10^{-5}$
Ler								
	RW	45	2	39,2	$1x10^{-5}$	2	36.2	$1x10^{-5}$
	IW	45	2	213.3	$1x10^{-5}$	2	646.7	$1x10^{-5}$
	SW	45	2	17.4	$1x10^{-5}$	2	59.5	$1x10^{-5}$

Accessions and traits (***RW:*** Rosette Weight; ***IW:*** Inflorescence Weight; ***SW:*** Seed Weight) are listed on the left. ***n*****:** number of observations. ***df*****:** degrees of freedom. ***F*****:** *F*-value from the type III sum of squares ANOVA for each factor. ***P:*** Estimated probability of obtaining this *F*-value under the null hypothesis.
