# Supplementary material for: Differential Tolerance to Direct and Indirect Density-Dependent Costs of Viral Infection in Arabidopsis thaliana
Source: PLoS Pathog. 2009 Jul 31;5(7):e1000531. doi: 10.1371/journal.ppat.1000531 (PMC2712083; doi:10.1371/journal.ppat.1000531)
Supplement: Table S4 — Three-way ANOVAs of life-history traits on Arabidopsis monocultures of infected (I) and mock-inoculated (M) plants, by using “plant condition (infected, I or mock-inoculated, M)”, “plant density” and “accession” as factors. (0.03 MB PDF) [file ppat.1000531.s005.pdf]

Table S4. Three-way ANOVAs of life-history traits on *Arabidopsis* monocultures of infected (I) and mock-inoculated (M) plants, by using "plant condition (infected, I or mock-inoculated, M)", "plant density" and "accession" as factors.

		**Plant condition**			**Plant Density**			**Accession**		
Trait	*n*	*df*	*F*	*P*	*df*	*F*	*P*	*df*	*F*	*P*
RW	290	1	16	$1x10^{-4}$	2	63.06	$1x10^{-5}$	2	498.34	$1x10^{-5}$
IW	290	1	11.73	$7x10^{-4}$	2	81.03	$1x10^{-5}$	2	41.10	$1x10^{-5}$
SW	290	1	75.54	$1x10^{-5}$	2	126.11	$1x10^{-5}$	2	174.77	$1x10^{-5}$

		**C x D**			**C x A**			**D x A**			**C x D x A**		
	n	*df*	*F*	*P*	*df*	*F*	*P*	*df*	*F*	*P*	*df*	*F*	*P*
RW	290	2	4.24	0.015	2	9.8	$1x10^{-4}$	4	46.44	$1x10^{-5}$	4	2.44	0.047
IW	290	2	0.65	0.524	2	0.34	0.712	4	4.99	$7x10^{-4}$	4	0.72	0.576
SW	290	2	24.38	$1x10^{-5}$	2	25.14	$1x10^{-5}$	4	7.84	$1x10^{-5}$	4	14.6	$1x10^{-5}$

Traits (***RW:*** Rosette Weight; ***IW:*** Inflorescence Weight; ***SW:*** Seed Weight) are listed on the left. ***n*****:** number of observations. ***df*****:** degrees of freedom. ***F*****:** *F*-value from the type III sum of squares ANOVA for each factor. ***P:*** Estimated probability of obtaining this *F*-value under the null hypothesis.
