# Supplementary material for: Differential Tolerance to Direct and Indirect Density-Dependent Costs of Viral Infection in Arabidopsis thaliana
Source: PLoS Pathog. 2009 Jul 31;5(7):e1000531. doi: 10.1371/journal.ppat.1000531 (PMC2712083; doi:10.1371/journal.ppat.1000531)
Supplement: Table S5 — One-way ANOVAs of the direct cost of CMV infection on Arabidopsis life-history traits. Comparison between monocultures of infected and mock-inoculated plants at each plant density. (0.03 MB PDF) [file ppat.1000531.s006.pdf]

Table S5. One-way ANOVAs of the direct cost of CMV infection on *Arabidopsis* life-history traits. Comparison between monocultures of infected and mock-inoculated plants at each plant density.

		**1 Plant**				**2 Plants**				**4 Plants**			
Accession	Trait	*n*	*df*	*F*	*P*	*n*	*df*	*F*	*P*		*df*	*F*	*P*
Boa-0													
	RW	30	1	8.85	0.005	30	1	8.20	0.007	30	1	7.93	0.008
	IW	30	1	0.04	0.841	30	1	0.30	0.619	30	1	7.4	0.010
	SW	30	1	0.03	0.873	30	1	5.01	0.032	30	1	8.2	0.007
Cen-1													
	RW	30	1	5.54	0.026	30	1	4.81	0.043	30	1	8.82	0.006
	IW	30	1	4.46	0.041	30	1	7.29	0.012	30	1	9.06	0.004
	SW	30	1	69.57	$1x10^{-5}$	30	1	5.13	0.036	30	1	5.24	0.031
Ler													
	RW	30	1	12.48	0.001	30	1	4.37	0.046	30	1	18.51	$2x10^{-4}$
	IW	30	1	138.1	$1x10^{-5}$	30	1	98.96	$1x10^{-5}$	30	1	53.43	$1x10^{-5}$
	SW	30	1	80.25	$1x10^{-5}$	30	1	5.57	0.029	30	1	4.78	0.045

Accessions and traits (***RW:*** Rosette Weight; ***IW:*** Inflorescence Weight; ***SW:*** Seed Weight) are listed on the left. ***n*****:** number of observations. ***df*****:** degrees of freedom. ***F*****:** *F*-value from the type III sum of squares ANOVA for each factor. ***P:*** Estimated probability of obtaining this *F*-value under the null hypothesis.
