# Supplementary material for: Differential Tolerance to Direct and Indirect Density-Dependent Costs of Viral Infection in Arabidopsis thaliana
Source: PLoS Pathog. 2009 Jul 31;5(7):e1000531. doi: 10.1371/journal.ppat.1000531 (PMC2712083; doi:10.1371/journal.ppat.1000531)
Supplement: Table S6 — Two-way ANOVAs of the direct cost of CMV infection (Traiti/Traitm) on Arabidopsis life-history traits, by using “plant density” and “accession” as factors. (0.02 MB PDF) [file ppat.1000531.s007.pdf]

Table S6. Two-way ANOVAs of the direct cost of CMV infection ($Trait_i$ /$Trait_m$) on *Arabidopsis* life-history traits, by using "plant density" and "accession" as factors.

		**Plant Density**			**Accession**			**D x A**		
Trait	*n*	*df*	*F*	*P*	*df*	*F*	*P*	*df*	*F*	*P*
RW_i/RW_m	135	2	13.62	1×10^{-5}	2	1.85	0.161	4	1.22	0.307
IW_i/IW_m	135	2	4.02	0.031	2	2.63	0.076	4	2.11	0.083
SW_i/SW_m	135	2	4.88	0.019	2	4.13	0.026	4	7.94	1×10^{-5}

Traits (**RW_i/RW_m:** Effect of CMV infection in Rosette Weight; **IW_i/IW_m:** Effect of CMV infection in Inflorescence Weight; **SW_i/SW_m:** Effect of infection in Seed Weight) are listed on the left. ***n*:** number of observations. ***df*:** degrees of freedom. ***F*:** *F*-value from the type III sum of squares ANOVA for each factor. ***P*:** Estimated probability of obtaining this *F*-value under the null hypothesis.
