# Supplementary material for: Differential Tolerance to Direct and Indirect Density-Dependent Costs of Viral Infection in Arabidopsis thaliana
Source: PLoS Pathog. 2009 Jul 31;5(7):e1000531. doi: 10.1371/journal.ppat.1000531 (PMC2712083; doi:10.1371/journal.ppat.1000531)
Supplement: Table S7 — One-way ANOVAs of the impact of host plant density and the direct cost of CMV infection (Traiti/Traitm) on Arabidopsis life-history traits. (0.02 MB PDF) [file ppat.1000531.s008.pdf]

Table S7. One-way ANOVAs of the impact of host plant density and the direct cost of CMV infection (*$Trait_i$ /$Trait_m$*) on *Arabidopsis* life-history traits.

		Plant Density			
Accession	Trait	*n*	*df*	*F*	*P*
Boa-0					
	RW_i/RW_m	45	2	2.33	0.108
	IW_i/IW_m	45	2	1.48	0.237
	SW_i/SW_m	45	2	6.89	0.002
Cen-1					
	RW_i/RW_m	45	2	7.61	0.015
	IW_i/IW_m	45	2	1.08	0.348
	SW_i/SW_m	45	2	4.88	0.027
Ler					
	RW_i/RW_m	45	2	11.53	1×10^{-4}
	IW_i/IW_m	45	2	213.28	1×10^{-5}
	SW_i/SW_m	45	2	6.61	0.003

Accessions and traits (***RW_i/RW_m:*** Effect of CMV infection in Rosette Weight; ***IW_i/IW_m:*** Effect of CMV infection in Inflorescence Weight; ***SW_i/SW_m:*** Effect of infection in Seed Weight) are listed on the left. ***n*****:** number of observations. ***df*****:** degrees of freedom. ***F*****:** *F*-value from the type III sum of squares ANOVA for each factor. ***P*****:** Estimated probability of obtaining this *F*-value under the null hypothesis.
