# Supplementary material for: Differential Tolerance to Direct and Indirect Density-Dependent Costs of Viral Infection in Arabidopsis thaliana
Source: PLoS Pathog. 2009 Jul 31;5(7):e1000531. doi: 10.1371/journal.ppat.1000531 (PMC2712083; doi:10.1371/journal.ppat.1000531)
Supplement: Table S8 — Three-way ANOVAs for the indirect cost of CMV infection in Arabidopsis life-history traits in infected and mock-inoculated plants, by using “class of competition”, “plant density” and “accession” as factors. (0.04 MB PDF) [file ppat.1000531.s009.pdf]

Table S8. Three-way ANOVAs for the indirect cost of CMV infection in *Arabidopsis* life-history traits in infected and mock-inoculated plants, by using "class of competition", "plant density" and "accession" as factors.

			**Class of competition**			**Plant Density**			**Accession**		
Plant Condition	Trait	*n*	*df*	*F*	*P*	*df*	*F*	*P*	*df*	*F*	*P*
Infected											
	RW	315	1	14.92	$1x10^{-4}$	1	11.42	$8x10^{-4}$	2	570.45	$1x10^{-5}$
	IW	315	1	14.05	$2x10^{-4}$	1	23.03	$1x10^{-5}$	2	32.80	$1x10^{-5}$
	SW	315	1	11.08	0.001	1	19.22	$1x10^{-5}$	2	68.83	$1x10^{-5}$
Mock-inoculated											
	RW	315	1	0.73	0.395	1	28.21	$1x10^{-5}$	2	514.57	$1x10^{-5}$
	IW	315	1	1.69	0.195	1	41.23	$1x10^{-5}$	2	25.65	$1x10^{-5}$
	SW	315	1	0.44	0.509	1	49.24	$1x10^{-5}$	2	166.09	$1x10^{-5}$

			**Cs x D**			**Cs x A**			**D x A**			**Cs x D x A**		
Plant Condition	Trait	*N*	*df*	*F*	*P*	*df*	*F*	*P*	*df*	*F*	*P*	*df*	*F*	*P*
Infected														
	RW	315	1	3.00	0.084	2	6.44	0.002	2	2.82	0.069	2	0.88	0.416
	IW	315	1	4.98	0.026	2	0.77	0.462	2	2.67	0.071	2	1.39	0.250
	SW	315	1	0.07	0.789	2	4.20	0.016	2	0.32	0.730	2	0.08	0.925
Mock-inoculated														
	RW	315	1	2.02	0.156	2	0.38	0.682	2	10.79	$1x10^{-5}$	2	1.64	0.195
	IW	315	1	0.07	0.788	2	1.81	0.166	2	1.55	0.214	2	0.80	0.450
	SW	315	1	1.64	0.201	2	0.92	0.401	2	0.03	0.971	2	1.61	0.281

Plant condition (I or M) and traits (***RW:*** Rosette Weight; ***IW:*** Inflorescence Weight; ***SW:*** Seed Weight) are listed on the left. Classes of competition are **IntraClass**: I, I/I, I/I/I/I and M, M/M, M/M/M/M; or **InterClass**: M/M/M/I, M/M/I/I, M/I/M/I, M/I/I/I. ***n*****:** number of observations. ***df*****:** degrees of freedom. ***F*****:** *F*-value from the type III sum of squares ANOVA for each factor. ***P:*** Estimated probability of obtaining this *F*-value under the null hypothesis.
