# Supplementary material for: Differential Tolerance to Direct and Indirect Density-Dependent Costs of Viral Infection in Arabidopsis thaliana
Source: PLoS Pathog. 2009 Jul 31;5(7):e1000531. doi: 10.1371/journal.ppat.1000531 (PMC2712083; doi:10.1371/journal.ppat.1000531)
Supplement: Table S9 — One-way ANOVAs for the indirect cost of CMV infection on Arabidopsis life-history traits in infected and mock-inoculated plants.Comparison between intra and interclass treatments at each plant density. (0.04 MB PDF) [file ppat.1000531.s010.pdf]

Table S9. One-way ANOVAs for the indirect cost of CMV infection on *Arabidopsis* life-history traits in infected and mock-inoculated plants. Comparison between intra and interclass treatments at each plant density.

			**Plant Density**							
			**2 Plants**				**4 Plants**			
Accession	Plant Condition	Trait	*df*	*n*	*F*	*P*	*n*	*df*	*F*	*P*
**Boa-0**										
	Infected									
		RW	1	30	5.98	0.020	75	4	1.50	0.210
		IW	1	30	4.64	0.045	75	4	0.15	0.963
		SW	1	30	0.02	0.889	75	4	1.11	0.360
	Mock-inoculated									
		RW	1	30	0.88	0.353	75	4	1.17	0.329
		IW	1	30	0.01	0.932	75	4	0.84	0.503
		SW	1	30	0.09	0.762	75	4	2.99	0.024
**Cen-1**										
	Infected									
		RW	1	30	6.97	0.013	75	4	0.83	0.512
		IW	1	30	5.64	0.025	75	4	3.43	0.015
		SW	1	30	8.13	0.008	75	4	3.10	0.021
	Mock-inoculated									
		RW	1	30	1.51	0.230	75	4	5.50	$7x10^{-4}$
		IW	1	30	0.67	0.419	75	4	11.28	$1x10^{-5}$
		SW	1	30	1.32	0.261	75	4	2.80	0.033
**L*er***										
	Infected									
		RW	1	30	65.20	$1x10^{-5}$	75	4	1.18	0.327
		IW	1	30	92.65	$1x10^{-5}$	75	4	20.26	$1x10^{-5}$
		SW	1	30	4.64	0.047	75	4	37.53	$1x10^{-5}$
	Mock-inoculated									
		RW	1	30	2.51	0.124	75	4	60.33	$1x10^{-5}$
		IW	1	30	0.14	0.707	75	4	11.71	$1x10^{-5}$
		SW	1	30	0.51	0.479	75	4	5.02	0.001

Accessions, plant condition (I or M) and traits (***RW:*** Rosette Weight; ***IW:*** Inflorescence Weight; ***SW:*** Seed Weight) are listed on the left. ***n*:** number of observations. ***df*:** degrees of freedom. ***F*:** *F*-value from the type III sum of squares ANOVA for each factor. ***P:*** Estimated probability of obtaining this *F*-value under the null hypothesis.
