# Supplementary material for: Differential Tolerance to Direct and Indirect Density-Dependent Costs of Viral Infection in Arabidopsis thaliana
Source: PLoS Pathog. 2009 Jul 31;5(7):e1000531. doi: 10.1371/journal.ppat.1000531 (PMC2712083; doi:10.1371/journal.ppat.1000531)
Supplement: Table S10 — Three-way ANOVAs of the effect of CMV infection (Traiti/Traitm) on Arabidopsis life-history traits, by using “class of competition”, “plant density” and “accession” as factors. (0.03 MB PDF) [file ppat.1000531.s011.pdf]

Table S10. Three-way ANOVAs of the effect of CMV infection (*$Trait_i$ /$Trait_m$*) on *Arabidopsis* life-history traits, by using "class of competition", "plant density" and "accession" as factors.

		Class of competition			Plant Density			Accession		
Trait	*n*	*df*	*F*	*P*	*df*	*F*	*P*	*df*	*F*	*P*
RW_i/RW_m	315	1	131.02	$1x10^{-5}$	1	13.30	$3x10^{-4}$	2	9.80	$1x10^{-4}$
IW_i/IW_m	315	1	27.29	$1x10^{-5}$	1	0.52	0.470	2	3.33	0.037
SW_i/SW_m	315	1	7.97	0.005	1	0.04	0.836	2	5.43	0.018

		Cs x D			Cs x A			D x A			Cs x D x A		
Trait	*n*	*df*	*F*	*P*	*df*	*F*	*P*	*df*	*F*	*P*	*df*	*F*	*P*
RW_i/RW_m	315	1	0.11	0.745	2	9.40	$1x10^{-4}$	2	0.07	0.935	2	6.76	0.005
IW_i/IW_m	315	1	1.15	0.284	2	3.27	0.033	2	0.44	0.645	2	3.81	0.023
SW_i/SW_m	315	1	0.09	0.767	2	7.43	$7x10^{-4}$	2	0.61	0.545	2	2.93	0.049

Traits (***RW_i/RW_m*:** Effect of CMV infection in Rosette Weight; ***IW_i/IW_m*:** Effect of CMV infection in Inflorescence Weight; ***SW_i/SW_m*:** Effect of infection in Seed Weight) are listed on the left. Classes of competition are **IntraClass**: I, I/I, I/I/I/I and M, M/M, M/M/M/M; or **InterClass**: M/M/M/I, M/M/I/I, M/I/M/I, M/I/I/I. ***n*:** number of observations. ***df*:** degrees of freedom. ***F*:** *F*-value from the type III sum of squares ANOVA for each factor. ***P*:** Estimated probability of obtaining this *F*-value under the null hypothesis.
