# Supplementary material for: Differential Tolerance to Direct and Indirect Density-Dependent Costs of Viral Infection in Arabidopsis thaliana
Source: PLoS Pathog. 2009 Jul 31;5(7):e1000531. doi: 10.1371/journal.ppat.1000531 (PMC2712083; doi:10.1371/journal.ppat.1000531)
Supplement: Table S11 — One-way ANOVAs of the effect of CMV infection (Traiti/Traitm) on Arabidopsis life-history traits. Comparison between intra and interclass treatments at each plant density. (0.03 MB PDF) [file ppat.1000531.s012.pdf]

Table S11. One-way ANOVAs of the effect of CMV infection ($Trait_i$ /$Trait_m$) on *Arabidopsis* life-history traits. Comparison between intra and interclass treatments at each plant density.

		Plant Density							
		2 Plants				4 Plants			
Accession	Trait	*n*	*df*	*F*	*P*	*n*	*df*	*F*	*P*
Boa-0									
	RW_i/RW_m	30	1	3.93	0.043	75	1	10.72	0.002
	IW_i/IW_m	30	1	4.88	0.037	75	1	0.25	0.619
	SW_i/SW_m	30	1	0.01	0.931	75	1	9.52	0.004
Cen-1									
	RW_i/RW_m	30	1	15.91	$4x10^{-4}$	75	1	26.22	$1x10^{-5}$
	IW_i/IW_m	30	1	12.27	0.002	75	1	35.86	$1x10^{-5}$
	SW_i/SW_m	30	1	6.55	0.022	75	1	16.08	$1x10^{-4}$
Ler									
	RW_i/RW_m	30	1	78.05	$1x10^{-5}$	75	1	13.82	$4x10^{-4}$
	IW_i/IW_m	30	1	100.62	$1x10^{-5}$	75	1	80.66	$1x10^{-5}$
	SW_i/SW_m	30	1	6.36	0.023	75	1	9.67	0.003

Accessions and traits (***RW_i/RW_m*:** Effect of CMV infection in Rosette Weight; ***IW_i/IW_m*:** Effect of CMV infection in Inflorescence Weight; ***SW_i/SW_m*:** Effect of infection in Seed Weight) are listed on the left. ***n*:** number of observations. ***df*:** degrees of freedom. ***F*:** *F*-value from the type III sum of squares ANOVA foreach factor. ***P:*** Estimated probability of obtaining this *F*-value under the null hypothesis.
