# Supplementary material for: Differential Tolerance to Direct and Indirect Density-Dependent Costs of Viral Infection in Arabidopsis thaliana
Source: PLoS Pathog. 2009 Jul 31;5(7):e1000531. doi: 10.1371/journal.ppat.1000531 (PMC2712083; doi:10.1371/journal.ppat.1000531)
Supplement: Table S12 — Two-way ANOVAs of the impact of host plant density in the indirect cost of CMV infection [(Traiti/Traitm)Inter-class/(Traiti/Traitm)Intra-class] on Arabidopsis life-history, by using “plant density” and “accession” as factors. (0.02 MB PDF) [file ppat.1000531.s013.pdf]

Table S12. Two-way ANOVAs of the impact of host plant density in the indirect cost of CMV infection $[(Trait_i/Trait_m)_{Inter\text{-}class}/(Trait_i/Trait_m)_{Intra\text{-}class}]$ on *Arabidopsis* life-history, by using "plant density" and "accession" as factors.

		Plant Density			Accession			D x A		
Trait	*n*	*df*	*F*	*P*	*df*	*F*	*P*	*df*	*F*	*P*
**Ratio *RW***	225	1	2.25	0.135	2	24.52	1×10^{-5}	2	5.80	0.004
**Ratio *IW***	225	1	3.38	0.067	2	4.63	0.011	2	6.78	0.001
**Ratio *SW***	225	1	0.49	0.485	2	13.28	1×10^{-5}	2	5.17	0.006

Traits (**Ratio *RW:*** $(RW_i/RW_m)_{Inter\text{-}class}/(RW_i/RW_m)_{Intra\text{-}class}$; **Ratio *IW:*** $(IW_i/IW_m)_{Inter\text{-}class}/(IW_i/IW_m)_{Intra\text{-}class}$; **Ratio *SW:*** $(SW_i/SW_m)_{Inter\text{-}class}/(SW_i/SW_m)_{Intra\text{-}class}$) are listed on the left. ***n*:** number of observations. ***df*:** degrees of freedom. ***F*:** *F*-value from the type III sum of squares ANOVA for each factor. ***P:*** Estimated probability of obtaining this *F*-value under the null hypothesis.
