# Supplementary material for: Differential Tolerance to Direct and Indirect Density-Dependent Costs of Viral Infection in Arabidopsis thaliana
Source: PLoS Pathog. 2009 Jul 31;5(7):e1000531. doi: 10.1371/journal.ppat.1000531 (PMC2712083; doi:10.1371/journal.ppat.1000531)
Supplement: Table S13 — One-way ANOVAs of the impact of host plant density in the indirect cost of CMV infection [(Traiti/Traitm)Inter-class/(Traiti/Traitm)Intra-class] on Arabidopsis life-history traits. (0.03 MB PDF) [file ppat.1000531.s014.pdf]

Table S13. One-way ANOVAs of the impact of host plant density in the indirect cost of CMV infection [$(Trait_i/Trait_m)_{Inter\text{-}class}/(Trait_i/Trait_m)_{Intra\text{-}class}$] on *Arabidopsis* life-history traits.

		Plant Density			
Accession	Trait	*n*	*df*	*F*	*P*
Boa-0					
	**Ratio *RW***	75	1	0.78	0.379
	**Ratio *IW***	75	1	7.06	0.009
	**Ratio *SW***	75	1	4.33	0.041
Cen-1					
	**Ratio *RW***	75	1	4.18	0.040
	**Ratio *IW***	75	1	0.01	0.924
	**Ratio *SW***	75	1	6.15	0.025
Ler					
	**Ratio *RW***	75	1	55.57	$1x10^{-5}$
	**Ratio *IW***	75	1	2.39	0.127
	**Ratio *SW***	75	1	4.17	0.039

Accessions and traits (**Ratio *RW:*** $(RW_i/RW_m)_{Inter\text{-}class}/(RW_i/RW_m)_{Intra\text{-}class}$; **Ratio *IW:*** $(IW_i/IW_m)_{Inter\text{-}class}/(IW_i/IW_m)_{Intra\text{-}class}$; **Ratio *SW:*** $(SW_i/SW_m)_{Inter\text{-}class}/(SW_i/SW_m)_{Intra\text{-}class}$) are listed on the left. ***n*****:** number of observations. ***df*****:** degrees of freedom. ***F*****:** *F*-value from the type III sum of squares ANOVA for each factor. ***P:*** Estimated probability of obtaining this *F*-value under the null hypothesis.
