# Supplementary material for: Differential Tolerance to Direct and Indirect Density-Dependent Costs of Viral Infection in Arabidopsis thaliana
Source: PLoS Pathog. 2009 Jul 31;5(7):e1000531. doi: 10.1371/journal.ppat.1000531 (PMC2712083; doi:10.1371/journal.ppat.1000531)
Supplement: Table S14 — Two-way ANOVAs of the impact of CMV prevalence at 4 plants per pot in the effect of CMV infection (Traiti/Traitm) on Arabidopsis life-history traits, by using “prevalence” and “accession” as factors. (0.02 MB PDF) [file ppat.1000531.s015.pdf]

Table S14. Two-way ANOVAs of the impact of CMV prevalence at 4 plants per pot in the effect of CMV infection (*$Trait_i$ /$Trait_m$*) on *Arabidopsis* life-history traits, by using “prevalence” and “accession” as factors.

Trait		Prevalence			Accession			P x A		
	n	*df*	*F*	*P*	*df*	*F*	*P*	*df*	*F*	*P*
RW_i/RW_m	225	4	21.97	$1x10^{-5}$	2	15.24	$1x10^{-5}$	8	2.12	0.045
IW_i/IW_m	225	4	3.14	0.016	2	8.94	$2x10^{-4}$	8	2.15	0.033
SW_i/SW_m	225	4	1.57	0.184	2	1.36	0.259	8	2.48	0.014

Traits (***RW_i/RW_m:*** Effect of CMV infection in Rosette Weight; ***IW_i/IW_m:*** Effect of CMV infection in Inflorescence Weight; ***SW_i/SW_m:*** Effect of infection in Seed Weight) are listed on the left. ***n*****:** number of observations. ***df*****:** degrees of freedom. ***F*****:** *F*-value from the type III sum of squares ANOVA for each factor. ***P:*** Estimated probability of obtaining this *F*-value under the null hypothesis.
