# Supplementary material for: Differential Tolerance to Direct and Indirect Density-Dependent Costs of Viral Infection in Arabidopsis thaliana
Source: PLoS Pathog. 2009 Jul 31;5(7):e1000531. doi: 10.1371/journal.ppat.1000531 (PMC2712083; doi:10.1371/journal.ppat.1000531)
Supplement: Table S15 — One-way ANOVAs of the impact of CMV prevalence at 4 plants per pot in the effect of CMV infection (Traiti/Traitm) on Arabidopsis life-history traits. (0.03 MB PDF) [file ppat.1000531.s016.pdf]

Table S15. One-way ANOVAs of the impact of CMV prevalence at 4 plants per pot in the effect of CMV infection ($Trait_i$ /$Trait_m$) on *Arabidopsis* life-history traits.

		Prevalence			
Accession	Trait	*n*	*df*	*F*	*P*
Boa-0					
	RW_i/RW_m	75	4	0.46	0.762
	IW_i/IW_m	75	4	2.17	0.113
	SW_i/SW_m	75	4	4.17	0.004
Cen-1					
	RW_i/RW_m	75	4	7.63	$1x10^{-5}$
	IW_i/IW_m	75	4	9.24	$1x10^{-5}$
	SW_i/SW_m	75	4	1.08	0.734
Ler					
	RW_i/RW_m	75	4	42.21	$1x10^{-5}$
	IW_i/IW_m	75	4	20.26	$1x10^{-5}$
	SW_i/SW_m	75	4	2.43	0.056

Accessions and traits (**RW_i/RW_m:** Effect of CMV infection in Rosette Weight; **IW_i/IW_m:** Effect of CMV infection in Inflorescence Weight; **SW_i/SW_m:** Effect of infection in Seed Weight) are listed on the left. ***n*:** number of observations. ***df*:** degrees of freedom. ***F*:** *F*-value from the type III sum of squares ANOVA for each factor. ***P*:** Estimated probability of obtaining this *F*-value under the null hypothesis.
