# Supplementary material for: Differential Tolerance to Direct and Indirect Density-Dependent Costs of Viral Infection in Arabidopsis thaliana
Source: PLoS Pathog. 2009 Jul 31;5(7):e1000531. doi: 10.1371/journal.ppat.1000531 (PMC2712083; doi:10.1371/journal.ppat.1000531)
Supplement: Table S16 — Three-way ANOVAs of SW/RW ratio, by using “plant condition (infected, I or mock-inoculated, M)”, “plant density” and “accession” as factors. (0.02 MB PDF) [file ppat.1000531.s017.pdf]

Table S16. Three-way ANOVAs of *SW/RW* ratio, by using “plant condition (infected, I or mock-inoculated, M)”, “plant density” and “accession” as factors.

		**Plant condition**			**Plant Density**			**Accession**		
Trait	*n*	*df*	*F*	*P*	*df*	*F*	*P*	*df*	*F*	*P*
SW/RW	720	1	3.92	0.045	2	8.71	2×10^{-4}	2	196.46	1×10^{-5}

		**C x D**			**C x A**			**D x A**			**C x D x A**		
Trait	*n*	*df*	*F*	*P*	*df*	*F*	*P*	*df*	*F*	*P*	*df*	*F*	*P*
SWRW	720	2	3.32	0.039	2	0.07	0.934	4	13.71	1×10^{-5}	4	1.12	0.344

Traits (***SW/RW:*** Seed Weight to Rosette Weight ratio) are listed on the left. ***n*****:** number of observations. ***df*****:** degrees of freedom. ***F*****:** *F*-value from the type III sum of squares ANOVA for each factor. ***P:*** Estimated probability of obtaining this *F*-value under the null hypothesis.
