# Supplementary material for: Differential Tolerance to Direct and Indirect Density-Dependent Costs of Viral Infection in Arabidopsis thaliana
Source: PLoS Pathog. 2009 Jul 31;5(7):e1000531. doi: 10.1371/journal.ppat.1000531 (PMC2712083; doi:10.1371/journal.ppat.1000531)
Supplement: Table S17 — One-way ANOVAs of SW/RW ratio in Arabidopsis accessions. Comparison between infected (I) and mock-inoculated (M) plants at each plant density. (0.03 MB PDF) [file ppat.1000531.s018.pdf]

Table S17. One-way ANOVAs of *SW/RW* ratio in *Arabidopsis* accessions. Comparison between infected (I) and mock-inoculated (M) plants at each plant density.

		Boa-0				Cen-1				L*er*			
Nº Plants	Treatment	*n*	*df*	*F*	*P*	*n*	*df*	*F*	*P*	*n*	*df*	*F*	*P*
1 Plant													
	I	30	1	12.87	$1x10^{-5}$	30	1	6.96	$1x10^{-3}$	30	1	14.39	$1x10^{-5}$
2 Plants													
	M/I	30	1	1.59	0.216	30	1	6.46	0.015	30	1	5.97	0.021
	I/I	30	1	0.16	0.696	30	1	0.02	0.897	30	1	1.32	0.260
4 Plants													
	M/M/M/I	30	1	7.76	0.002	30	1	5.88	0.024	30	1	7.25	0.009
	M/M/I/I	30	1	6.32	0.018	30	1	0.15	0.704	30	1	0.04	0.844
	M/I/M/I	30	1	4.62	0.035	30	1	0.67	0.420	30	1	0.00	0.954
	M/I/I/I	30	1	0.06	0.802	30	1	4.21	0.042	30	1	5.91	0.024
	I/I/I/I	30	1	18.01	$1x10^{-5}$	30	1	0.80	0.498	30	1	6.10	0.019

Plant densities and treatments are listed on the left. ***n*****:** number of observations. ***df*****:** degrees of freedom. ***F*****:** *F*-value from the type III sum of squares ANOVA for each factor. ***P:*** Estimated probability of obtaining this *F*-value under the null hypothesis.
